# ALDH^High^ Breast Cancer Stem Cells Exhibit a Mesenchymal–Senescent Hybrid Phenotype, with Elevated Metabolic and Migratory Activities

**DOI:** 10.3390/cells13242059

**Published:** 2024-12-13

**Authors:** Luis Larrea Murillo, Conor J. Sugden, Bela Ozsvari, Zahra Moftakhar, Ghada S. Hassan, Federica Sotgia, Michael P. Lisanti

**Affiliations:** 1 Translational Medicine, School of Science, Engineering and Environment (SEE), University of Salford, Greater Manchester, Salford M5 4WT, UK; luis.larreamurillo@manchester.ac.uk (L.L.M.); bozsvari@lunellabiotech.com (B.O.); z.moftakhar@edu.salford.ac.uk (Z.M.); 2 Lunella Biotech, 1145 Carling Avenue, Ottawa, ON K1Z 7K4, Canada

**Keywords:** cancer stem cells, EMT, aldehyde dehydrogenase, cell senescence, metabolic reprogramming, mtDNA

## Abstract

Cancer stem cells (CSCs) account for 0.01 to 2% of the total tumor mass; however, they play a key role in tumor progression, metastasis and resistance to current cancer therapies. The generation and maintenance of CSCs are usually linked to the epithelial–mesenchymal transition (EMT), a dynamic process involved in reprogramming cancer cells towards a more aggressive and motile phenotype with increased stemness potential. Cells that undergo an EMT process have shown to be more resistant to conventional chemo/radiotherapies. In this context, aldehyde dehydrogenase (ALDH) enzymes, known for their role in the cellular detoxification of aldehydes and enhancement of cell survival, are often upregulated in cancer cells, promoting their resistance to conventional cancer treatments. Indeed, high ALDH levels have become a hallmark biomarker of CSCs and are often used to isolate this sub-population from the more abundant cancer cell populations. Herein, we isolated human breast cancer epithelial cells with higher ALDH abundance (ALDH^High^) and compared them to those with low ALDH abundance (ALDH^Low^). ALDH^High^ sub-populations exhibited more characteristic EMT biomarkers by adopting a more mesenchymal phenotype with increased stemness and enhanced migratory potential. Furthermore, ALDH^High^ sub-populations displayed elevated senescent markers. Moreover, these cells also demonstrated higher levels of mitochondria DNA/mass, as well as greater mitochondrial and glycolytic metabolic function. Conversely, ALDH^Low^ sub-populations showed a higher efficiency of mammosphere/colony formation and an increased proliferative capacity. Therefore, we demonstrated that these ALDH sub-populations have distinct characteristics, underscoring their role in EMT, the formation of tumors and the mechanisms of metastasis.

## 1. Introduction

The cancer stem cell (CSC) hypothesis proclaiming that tumors are sustained by a small number of cells with stemness properties is increasingly adopted as a model for tumor development, metastasis and resistance to therapy. Currently, breast cancer remains one of the most common cancer-related mortalities worldwide [1]. Cancer stem cells (CSCs) constitute approximately 0.01 to 2% of the total tumor mass [1,2]. Nevertheless, they are believed to be the main culprits for the inefficiency of current cancer therapies and have shown to be highly resistant to conventional chemo/radiotherapy strategies. In addition, an increasing amount of evidence has demonstrated their significant implication in the progression and metastasis of breast cancer tumors [3,4].

The epithelial–mesenchymal transition (EMT) is a dynamic process highly involved in cancer progression via reprogramming epithelial cells towards a more mesenchymal-like phenotype with increased stemness [5,6]. Interestingly, CSCs undertaking the EMT process are shown to exhibit increased CD44 expression and a high activity of aldehyde dehydrogenase (ALDH) enzymes, two biomarkers known for their high correlation with CSCs in breast cancer [7,8]. CD44, a hyaluronic acid transmembrane glycoprotein overexpressed by CSCs, is known for regulating the adhesion, survival, migration and motility of cancer cells. This promotes their escape or resistance to the effects of current drug/radiation therapeutic approaches and potentiates relapses and the metastasis of breast tumors [9,10]. ALDHs are a group of enzymes responsible for oxidizing aldehydes via Nicotinamide adenine dinucleotide phosphate (NADP)-dependent mechanisms and play a role in protecting cells against the deleterious effects of toxication and oxidative stress [11,12]. However, these cell-protective characteristics of ALDHs are key contributors to the resistance of tumor cells of various cancers to chemotherapeutic drugs. Therefore, high ALDH activity has become a hallmark characteristic of CSCs and is associated with their enhanced stemness, metastatic, migratory and invasive potential [12,13]. Consequently, the isolation of cancer cells with high ALDH levels has become a widely used marker for identifying CSC populations [14,15].

Another key biomarker associated with the EMT process is vimentin, a common marker of mesenchymal stem cells (MSCs) [16]. Vimentin is a cytoskeletal protein involved in focal adhesion for the extracellular attachment, migration, motility and overall structural support of cells [16,17]. CD44^+^/ALDH^High^ cancer cells were shown to exhibit activated vimentin, correlating with enhanced cell plasticity and tumorigenicity. Furthermore, cells that adopted a mesenchymal phenotype through the EMT process and expressed high levels of vimentin were shown to display significant metabolic reprogramming [7,18,19]. Therefore, these EMT biomarkers and metabolic reprogramming changes are believed to be important contributors to the therapeutic escape of CSCs [20,21].

While CSC markers have been well established and are known to be highly resistant to conventional cancer therapy, much is still unknown about those that survive and develop therapy-induced senescence. It has been shown that cells treated with Adriamycin, an anthracycline antibiotic used in chemotherapy, or radiotherapy could be induced into a senescent phenotype with enhanced stemness and ALDH activity [22,23]. However, the physiological consequence of elevated ALDH levels in CSCs is poorly understood. In this study, we segregated MDA-MB-231 epithelial cancer stem cells into ALDH^Low^ and ALDH^High^ sub-populations. These ALDH sub-populations demonstrated significantly distinct EMT properties including vimentin levels, stemness, metabolic activity, proliferation and migration. Moreover, differences in the colony/mammosphere formation capacity, cell cycle and cell senescence were also observed between these sub-populations. Our results reveal that while ALDH^High^ sub-populations possess increased cell plasticity and migratory behavior and higher levels of senescence markers compared to ALDH^low^ sub-populations, they displayed a lower proliferative capacity.

## 2. Materials and Methods

### 2.1. Cell Culture

Breast cancer epithelial cells MDA-MB-231, MCF7 and MDA-MB-468 were obtained commercially from the American Type Culture Collection (ATCC). Cells were cultured in Dulbecco’s Modified Eagle Medium (DMEM; Gibco, 41966029, USA) supplemented with 10% fetal bovine serum (FBS; Gibco, 10082-147, USA), 1X Glutamax (Gibco, 35050-061, USA) and 1% Penicillin–Streptomycin (Sigma-Aldrich, P0781, Poole, UK). Cells were maintained at 37 °C in a humidified air incubator containing carbon dioxide (5%). When cells reached ≃80% confluency, they were trypsinized, harvested, washed and used for experiments or passaged for further expansion.

### 2.2. FACS for Aldehyde Dehydrogenase (ALDH)-High and -Low Sub-Populations

**Figure 1:**
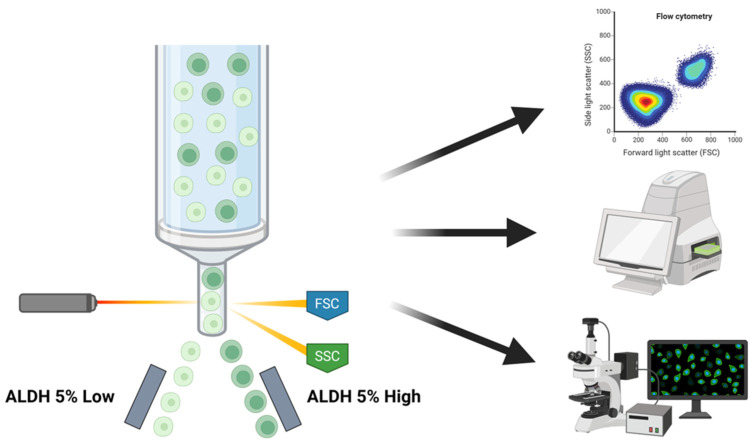
Schematics of cell sorting method with ALDEFLUOR™ Kit and subsequent experiments conducted to analyze differences in ALDH^Low^ and ALDH^High^ sub-populations.

MDA-MB-231 cells were sorted via Florescence-Associated Cell Sorting (FACS) using an ALDEFLUOR™ Kit (Stem Cell Technologies, 01700, Vancouver, BC, Canada). Cells were stained with the ALDEFLUOR™ Kit according to the manufacturer’s recommendations and then sorted using a Sony LE-SH800S Cell Sorter for cells displaying the 5% lowest fluorescence intensity (ALDH^Low^) and the 5% highest fluorescence intensity (ALDH^High^). The sub-populations were then analyzed via flow cytometry and various microplate assays and imaging techniques for characterization (Figure 1).

### 2.3. Lentiviral Transduction and FACS of Vimentin-Transfected Cells

MDA-MB-231 cells were transfected with RFP-Vimentin (Puro) Lentiviral particles (GeneTarget Inc, LVP1340-R, Abingdon, UK) according to the manufacturer’s protocol. After viral transduction, cell lines were selected with puromycin for two weeks. Thereafter, cells were sorted via FACS using the Sony LE-SH800S Cell Sorter for cells displaying the 5% lowest fluorescence intensity (Vimentin^Low^) and the 5% highest fluorescence intensity (Vimentin^High^). The sub-populations were then stained with the ALDEFLUOR™ Kit according to the manufacturer’s recommendations and analyzed via flow cytometry to determine the percentage of ALDH-positive cells within each sub-population.

### 2.4. Vimentin Analysis via Immunofluorescence Imaging and Flow Cytometry

Once sorted, cells were either seeded on 24-well plates at a density of 100,000 cells/well for immunofluorescence imaging or collected in cell pellets for flow cytometry analysis. Cells cultured on 24-well plates were fixed with 4% paraformaldehyde (PFA) 24 h post-sorting and permeabilized with 0.2% Triton X-100 prior to being blocked in 1% bovine serum albumin (BSA) for 1 h. Thereafter, cells were incubated with Vimentin primary Antibody V9 (AMSBIO, SC-6260, Cambridge, MA, USA) for 1h at RT and conjugated with an Alexa Fluor 660 anti-mouse secondary antibody (Invitrogen, A11012, Carlsbad, CA, USA) incubated for 1h at RT thereafter. Hoechst 33342, Trihydrochloride, Trihydrate (Invitrogen, H3570, Carlsbad, CA, USA) was counterstained to show nuclei. Cell pellets collected post-sorting were stained live with BioTracker™ TiY Vimentin Live Cell Dye (Sigma-Aldrich, SCT059, Saint Louis, MO, USA) for flow cytometry analysis and vimentin quantification using an Attune™ NxT flow cytometer (ThermoFisher Scientific, Waltham, MA, USA).

### 2.5. Immunofluorescence Imaging of mtDNA

Post-sorting, cells were seeded on 24-well plates for immunofluorescence imaging and fixed and permeabilized as detailed above. Thereafter, cells were blocked with 3% BSA for 45 min at RT and subsequently incubated with AC-30-10 mtDNA primary antibody (Progen, 690014, Heidelberg, Germany) for 1h at RT. The primary antibody was then conjugated with an Alexa Fluor 594 anti-mouse secondary antibody (Invitrogen, A11005, Carlsbad, CA, USA) incubated for 1h at RT and counterstained with Hoechst 33342 to show nuclei.

### 2.6. ATP Assay Using Cell-Titer-Glo 2.0

Cells sorted for ALDH^Low^ and ALDH^High^ sub-populations were seeded in 96-well plates at a density of 10,000 cells/well in DMEM supplemented with 10% FBS, 1X Glutmax and 1% Penicillin–Streptomycin. Cell-Titer-Glo 2.0 Reagent from Promega (G9242, Madison, WI, USA) was added to the culture media, and cells were incubated at 37 °C in a humidified air incubator containing carbon dioxide (5%) for 15 min. To quantify ATP, Luminescence content was evaluated using a Varioskan™ LUX plate reader (ThermoFisher Scientific, Waltham, MA, USA).

### 2.7. Flow Cytometry Analysis of Stemness Markers

After FACS, ALDH^Low^ and ALDH^High^ sub-populations were fixed with PFA 4% and stained for epithelial–mesenchymal transition (EMT)/MSC-associated stem markers (CD44, CD73, CD90, CD105) or pluripotent markers (OCT 4, SOX 2 and NANOG). Cells were washed and resuspended in 1% BSA PBS and analyzed via flow cytometry using the Attune™ NxT Flow Cytometer (ThermoFisher Scientific, Waltham, MA, USA). A list of all the antibodies used is displayed in Figure S1. SOX 2 unconjugated antibody was conjugated with an Alexa Fluor 594 anti-rabbit secondary antibody (Invitrogen, A11012, Carlsbad, CA, USA).

### 2.8. Seahorse XFe-96 Metabolic Flux Analysis

Extracellular acidification rates (ECARs) and real-time oxygen consumption rates (OCRs) for ALDH-expressing MDA-MB-231 sub-populations were determined using a Seahorse Extracellular Flux (XFe96) analyzer (Seahorse Bioscience, North Billerica, MA, USA). For analysis, 30,000 cells/well were seeded after sorting in XFe-96-well plates and incubated at 37 °C in a humidified air incubator containing carbon dioxide (5%) for 24 h. Thereafter, cells were maintained in 175 μL/well of XF assay media at 37 °C, in a non-CO_2_ incubator for 1 h. During the incubation period, 5 μL of 80 mM glucose, 9 μM oligomycin and 1 M 2-deoxyglucose (for ECAR measurement) or 10 μM oligomycin, 9 μM FCCP, 10 μM Rotenone and 10 μM antimycin A (for OCR measurement) were loaded in XF assay media into the injection ports in the XFe-96 sensor cartridge. Measurements were normalized to account for cell density by assessing the fluorescence intensity of cell nuclei stained with Hoechst 33342, Trihydrochloride, Trihydrate (Invitrogen, H3570, Carlsbad, CA, USA). Fluorescence was measured by the Varioskan™ LUX plate reader at an excitation/emission of 355/460. All experiments were performed three times independently. Data sets were analyzed by XFe-96 software (version 2.6.3.5).

### 2.9. Mammosphere Formation

Post-sorting, single-cell suspensions were seeded in 6-well plates under non-adherent conditions at a density of 500 cells/cm^2^. Tissue culture plates were previously coated with Poly 2-hydroxyethyl methacrylate (poly-HEMA, Sigma-Aldrich, P3932, Germany) to generate non-adherent surfaces, and cells were cultured in mammosphere medium consisting of the following: DMEM F12 (Gibco, 21041-025, Billings, MT, USA), 20ng/ml B27 (Gibco, 17504-044, USA) 20 ng/mL EGF (Peprotech, AF-100-15, Cranbury, NJ, USA) and 1% Penicillin–Streptomycin. Cells were grown for 7 days and maintained at 37 °C in a humidified air incubator containing carbon dioxide (5%). After incubation, mammospheres greater than 50 μm were counted using a graticule.

### 2.10. Colony Formation

After sorting, single-cell suspensions were seeded in 6-well tissue culture plates at a density of 25 cells/cm^2^. Cells were cultured in DMEM supplemented with 10% FBS, 1X Glutmax and 1% Penicillin–Streptomycin. Cells were grown for 14 days and maintained at 37 °C in a humidified air incubator containing carbon dioxide (5%). Media were replaced once after the first 7 days with fresh media. After 14 days, colonies were fixed with 100% methanol at 4 °C for 30 min and stained with 0.5% Crystal Violet for 10 min at RT. Stained cell colonies in each well were counted, and the results were representative of three independent experiments.

### 2.11. Lipofuscin Analysis for Cell Senescence

Post-sorting, ALDH-expressing sub-populations were analyzed for lipofuscin via immunofluorescence imaging and flow cytometry. To image cells, cells were seeded onto coverslips and incubated for 36 h prior to fixing with PFA 4%. Immunostaining was performed according to PAN-Biotech UK Ltd. (Wimborne, UK)’s instructions. Cells were washed with Ethanol prior to staining with SenTraGor™ and thereafter with the fluorescent-labeled secondary antibody Anti-biotin Fluorescein (Vector Laboratories, SA-5001-1, Newark, CA, USA). The coverslips were then mounted onto microscope slides by VECTASHIELD^®^ Antifade Mounting Medium with DAPI (H-1200), and images were taken with an EVOS FL Auto 2 microscope (ThermoFisher Scientific, Waltham, MA, USA). For flow cytometry analysis, cell pellets were collected after FACS and fixed with PFA 4%. Pellets were then stained in tubes using the same manufacturer’s (PAN-Biotech UK Ltd.’s) instructions detailed above and analyzed using the Attune™ NxT Flow Cytometer (ThermoFisher Scientific, Waltham, MA, USA).

### 2.12. β-Galactosidase Analysis for Cell Senescence

The detection and analysis of β-galactosidase for ALDH^Low^- and ALDH^High^-sorted MDA-MB-231 sub-populations were conducted using a Senescence β-Galactosidase Staining Kit (Cell Signaling Technology, 9860, Danvers, MA, USA) for imaging and a CellEvent™ Senescence Green Flow Cytometry Assay Kit (ThermoFisher Scientific, C10840, Waltham, MA, USA) for flow cytometry analysis. The Senescence β-Galactosidase Staining Kit was used according to the manufacturer’s instructions, and imaging was conducted using the EVOS FL Auto 2 microscope (ThermoFisher Scientific, Waltham, MA, USA). For flow cytometry analysis, cells were first fixed in pellets with PFA 4% and thereafter stained with the CellEvent™ Senescence Green Flow Cytometry Assay Kit per the manufacturer’s instructions. Thereafter, cells were analyzed using the Attune™ NxT Flow Cytometer (ThermoFisher Scientific, Waltham, MA, USA).

### 2.13. Cell Cycle Analysis

Post-sorting, cells were stained with propidium iodide using a Muse^®^ Cell Cycle Kit (Luminex, MCH100106, Seattle, WT, USA). Staining was performed according to the manufacturer’s instructions, and cells were analyzed via flow cytometry with the Attune™ NxT Flow Cytometer (ThermoFisher Scientific, Waltham, MA, USA).

### 2.14. Scratch Assay to Evaluate Cell Migration

Prior to sorting, Culture-Insert 2 Well inserts from Ibidi (81176, Fitchburg, WI, USA) were placed and prepared in 12-well plates. Thereafter, cells were sorted for ALDH^Low^ and ALDH^High^ sub-populations and seeded in the inserts at a cell density of 500,000 cells per insert. Cells were cultured in DMEM supplemented with 10% FBS, 1X Glutmax and 1% Penicillin–Streptomycin and allowed to adhere and grow overnight. Thereafter, inserts were removed, and the gaps of cell-free areas were measured at different time-points (0 h, 4 h, 8 h, 16 h, 24 h).

### 2.15. Sulphorhodamine B Assay

After FACS, the protein content of MDA-MB-231 ALDH^Low^ and ALDH^High^ sub-populations was assessed via a sulphorhodamine (SRB) assay. Cells were fixed with 10% trichloroacetic acid (TCA) for 1 h in the cold and dried overnight at RT. Cells were then incubated with SRB for 15 min, washed with 1% acetic acid and air-dried for at least 3 h or overnight. Lastly, protein-bound dye was dissolved in a 10 mM Tris pH 8.8 solution, and absorbance was read using the Varioskan™ LUX plate reader (Thermo Fisher Scientific, USA) at 562 nm.

### 2.16. Measurement of Lysosomal Mass

Post-sorting, the lysosomal mass of ALDH^Low^ and ALDH^High^ sub-populations was measured using LysoTracker™ Deep Red (Thermo Fisher Scientific, L12492, USA). Cells were collected as pellets and stained live with LysoTracker™ Deep Red. Fluorescence intensity was then analyzed via flow cytometry analysis using the Attune ™ NxT Flow Cytometer (ThermoFisher Scientific, USA).

### 2.17. RNA, DNA and qPCR

RNA was extracted using a Monarch Total RNA Miniprep Kit (New England Biolabs, Ipswich, MA, USA) following the manufacturer’s instructions for cultured mammalian cells, and samples were diluted to a final concentration of 20 ng/μL. DNA extractions were performed using a Monarch^®^ Genomic DNA Purification Kit (New England Biolabs) for cell pellets of 1 × 10^6^ cells. DNA was subsequently diluted to a final concentration of 5 ng/μL. The relative mitochondrial DNA (mt-DNA) copy number was obtained using a Relative Human Mitochondrial DNA Copy Number Quantification qPCR Assay Kit (ScienCell, Carlsbad, CA, USA). Briefly, the 10 μL reaction contained the following: 5 μL Reaction MasterMix, 1 μL primers (10 μM, Fwd + Rv mixed), 1 μL of DNA (5 ng) and 2.7 μL water. qPCRs were performed in triplicate, and three biological repeats were analyzed for each condition. qPCRs were performed in a StepOnePlus™ Real-Time PCR System (Applied Biosystems, Waltham, MA, USA) with conditions as follows: 95 °C for 10 min, followed by 32 cycles of 95 °C for 20 s, 52 °C for 20 s and 72 °C for 45 s. The relative mtDNA levels were calculated using the ΔΔCT method [24].

A Luna Universal One-Step RT-qPCR Kit (New England Biolabs) was used for RT-qPCRs. Briefly, the 10 μL reaction contained the following: 5 μL Reaction MasterMix, 0.5 μL EnzymeMix, 0.4 μL primers (10 μM, Fwd + Rv mixed), 1 μL of RNA (20 ng) and 3.1 μL water. RT-qPCRs were performed in triplicate, and three biological repeats were analyzed for each condition. RT-qPCRs were performed in a StepOnePlus™ Real-Time PCR System (Applied Biosystems) with conditions as follows: 55 °C for 10 min and 95 °C for 1 min, followed by 45 cycles of 95 °C for 10 s and 60 °C for 30 s. The cycle threshold (Ct) value was calculated for each sample and normalized to GAPDH. The relative expression levels were calculated using the ΔΔCT method [24]. Primer sequences and melting curves for all the primers used are displayed in Figure S2.

### 2.18. RNAseq Analysis

Total RNA was purified using a Monarch^®^ Total RNA Miniprep Kit (New England Biolabs) following the manufacturer’s instructions. Novogene’s (Novogene (UK) Company Limited, Cambridge, UK) RNA sequencing services were used for all mRNA sequencing. Briefly, an mRNA library was generated from total RNA, quantified and sequenced using the Illumina NovaSeq 6000 platform (Illumina, San Diego, CA, USA). Clean data were obtained from raw reads, and all downstream analyses were based on clean data. A reference genome index was built using Hisat2 v2.0.5. Differential expression analysis was completed using DESeq2, and *p*-values were adjusted using Benjamini and Hochberg’s approach for controlling the false discovery rate. Genes with an adjusted *p*-value ≤ 0.05 found by DESeq2 were assigned as differentially expressed. Enrichment analyses were completed using the clusterProfiler R package (version 4.14.4) to test the statistical enrichment of differential expression genes in the GO, KEGG, Reactome and DO databases. The Dataset for RNAseq analysis is publicly available via the Harvard Dataverse online data repository (https://doi.org/10.7910/DVN/LRFZWI (accessed on 9 December 2024)).

### 2.19. Azithromycin Treatment

ALDH^High/Low^ sub-populations sorted using FACS, a heterogenous unsorted population of MDA-MB-231, hTERT-BJ1 human immortalized fibroblast and MRC-5 human lung fibroblast cells were plated into 96-well plates (6000 cells/well) for 2 days before treatment. Azithromycin (PHR1088-1G, Merck Life Science UK Ltd., London, UK) or vehicle (DMSO) was added to the plates for 6 days. After incubation, the 96-well plates were washed with PBS and incubated with Hoechst 33342, Trihydrochloride, Trihydrate (Invitrogen, H3570, Carlsbad, CA, USA) for 30 min then read with a plate reader at an excitation/emission of 355/460 nm. Values then were normalized to control data, and all treatments were repeated three times unless stated otherwise.

### 2.20. Statistical Analysis

Data were analyzed using GraphPad Prism software (GraphPad Software Inc. Version 9.3.1, San Diego, CA, USA) and Microsoft Excel (Microsoft Corporation, Redmond, WT, USA). Statistical significance was determined using Student’s *t* test or an ANOVA test if more than two groups were compared. Data are shown as the mean ± SEM unless stated otherwise. All experiments were performed at least three times independently, with three or more technical replicates for each experimental condition tested, unless stated otherwise. Values of *p* < 0.05 were considered significant, where * *p* ≤ 0.05, ** *p* ≤ 0.01, *** *p* ≤ 0.001 and **** *p* ≤ 0.0001. *p* > 0.05 was considered not significant (ns).

## 3. Results

### 3.1. ALDH^High^ Sub-Populations Present with Different Morphologies and Increased Levels of Vimentin as Compared to ALDH^Low^ Cells

**Figure 2:**
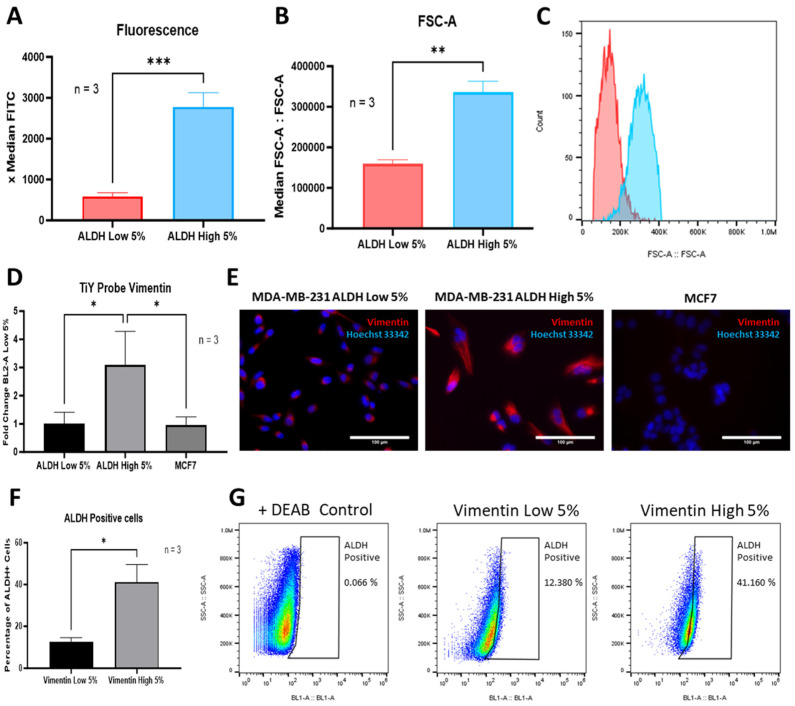
Cells were sorted for the 5% lowest fluorescence intensity and 5% highest fluorescence intensity to identify the ALDH^Low^ and ALDH^High^ sub-populations, respectively, of the heterogenous MDA-MB-231 population (**A**). Cell size was measured using the median FSC-A for the ALDH^High^ sub-populations and the ALDH^Low^ sub-populations (**B**,**C**). ALDH^High^ sub-populations were also shown to have higher protein levels of vimentin compared to ALDH^Low^ and MCF7 cells when measured via flow cytometry and visualized in immunofluorescence images (**D**,**E**). The percentage of ALDH-positive cells measured via flow cytometry was observed to be higher in Vimentin^High^-sorted cells compared to Vimentin^Low^-sorted cells (**F**,**G**). Experiments were performed at least 3 times independently. Data significance is presented as * *p* ≤ 0.05, ** *p* ≤ 0.01 and *** *p* ≤ 0.001.

Over the years, studies have observed differences in the cell size and stemness of cancer cells with contrasting results being reported [25,26]. The average dimension of an epithelial cell (~10–17 μm) is smaller than that of an MSC (~15–30 μm) [27,28,29]. Given that the EMT process commonly associated with CSCs drives their differentiation into a more mesenchymal phenotype, this might highly influence these cells to adopt a larger size. In our study, MDA-MB-231 cells were categorized for their expression of ALDH using FACS analysis (Figure 1). Cells were sorted for the 5% lowest fluorescence intensity and 5% highest fluorescence intensity to identify the ALDH^Low^ and ALDH^High^ sub-populations, respectively. Cells sorted for high levels of ALDH (Figure 2A) displayed a greater cell size as measured by their FSC-A electrical signal as compared to cells with low ALDH levels (Figure 2B,C). This morphological characteristic was also observed in MDA-MB-468 cells sorted for their levels of ALDH (Figure S3). Interestingly, these cells with a higher expression of ALDH, denominated as the ALDH^High^ sub-population, also demonstrated increased levels of vimentin (Figure 2D,E). In comparison, ALDH^Low^ sub-populations not only exhibited lower levels of vimentin, but their vimentin expression was also similar to that seen in the MCF7 cell line, known for its low vimentin content (Figure 2E). To further validate our results, MDA-MB-231 cells were transfected with a vimentin RFP promoter, selected as Vimentin^High^ or Vimentin^Low^ sub-populations and evaluated for ALDH expression. A higher frequency of ALDH-positive cells (41.106%) was observed in the Vimentin^High^-sorted cells compared to the Vimentin^Low^ sub-populations (12.380%) (Figure 2F,G). In addition, our Vimentin^High^ sub-populations displayed a larger size than Vimentin^Low^ cells, similarly to the size difference observed in ALDH^High^ vs. ALDH^Low^ cells but to a lesser degree (Figure S4). Thus, these results suggest that cells exhibiting high levels of ALDH adopted a mesenchymal phenotype or underwent an EMT process as compared to ALDH^Low^ sub-populations and potentially have an increased stemness potential.

### 3.2. ALDH^High^ Cells Were Shown to Possess a Mesenchymal Phenotype and Increased Markers of Pluripotency

**Figure 3:**
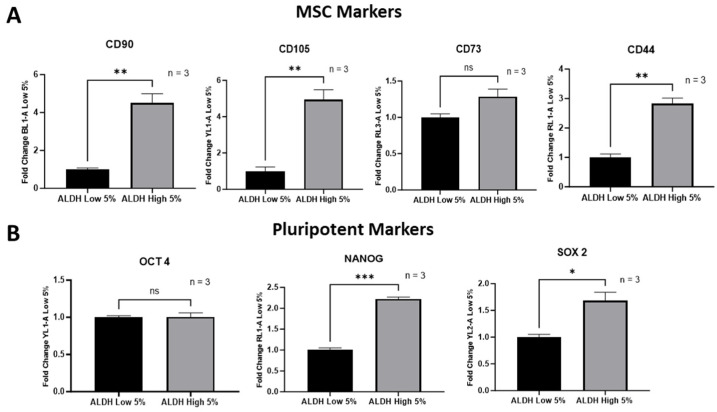
An increase in multiple MSC markers (CD90, CD015 and CD44) was observed via flow cytometry (**A**). Pluripotent markers NANOG and Sox 2 were also observed to increase as analyzed by flow cytometry (**B**). Experiments were performed at least 3 times independently. Data significance is presented as * *p* ≤ 0.05, ** *p* ≤ 0.01 and *** *p* ≤ 0.001. Value *p* > 0.05 were considered not significant (ns).

As mentioned above, various studies have demonstrated that CSCs are highly associated with the EMT process and thus are prone to adopting a more mesenchymal phenotype. In the context of EMT, pluripotency markers such as Nanog and Oct 4 have been shown to be involved in promoting EMT and the cell migration of breast CSCs [30,31,32]. By evaluating the markers of the mesenchymal transition in ALDH-expressing CSCs, our data show that ALDH^High^ sub-populations exhibited higher levels of MSC markers, CD90 and CD105, along with CD44, which is often associated with both MSCs and CSCs, as compared to ALDH^Low^ cells (Figure 3A). Pluripotency markers such as Nanog and Sox 2 were also shown to be upregulated in ALDH^High^ cells. However, other MSC/pluripotency markers including CD73 and Oct 4 appeared to be similarly expressed in both ALDH^High^ and ALDH^Low^ sub-populations (Figure 3B). These data further underscore the mesenchymal transition potential of ALDH^High^ CSCs.

### 3.3. ALDH^Low^ Sub-Populations Are More Adept at Forming Mammospheres and Colonies

**Figure 4:**
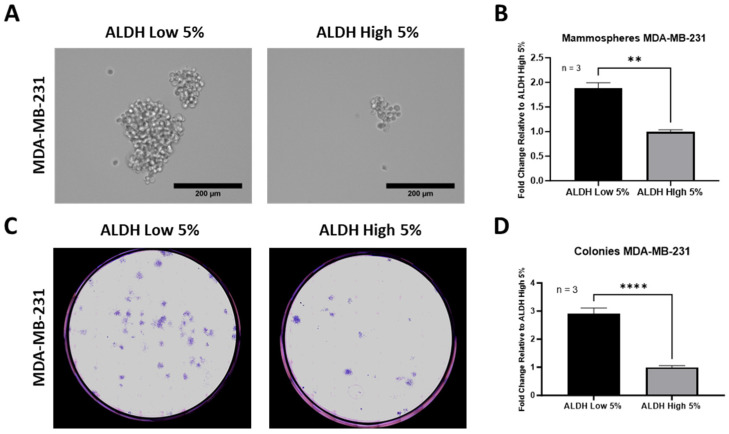
ALDH^Low^ MDA-MB-231 cells are more efficient at generating mammospheres and colonies. Mammosphere assay demonstrated that ALDH^Low^ sub-populations generated larger mammospheres at greater quantity than ALDH^High^ sub-populations (**A**,**B**). Similar results were observed in colony assay by even greater degree (**C**,**D**). Experiments were performed at least 3 times independently. Data significance is presented as ** *p* ≤ 0.01 and **** *p* ≤ 0.0001.

Various studies over the years have shown that cells with an increased expression of ALDH or even just ALDH-positive cells have a greater capacity for stemness and an ability to form mammospheres and colonies [19,33,34]. Thus, we assessed mammosphere/colony formation in our CSCs. To our surprise, the results demonstrated an opposite correlation between the number of generated mammospheres/colonies and the levels of expressed ALDH. ALDH^Low^ cells were capable of forming almost a 2-fold higher frequency of mammospheres after 7 days (40.00 ± 2.34) as compared to ALDH^High^ sub-populations (21.22 ± 0.8678), and their mammospheres were significantly larger (Figure 4A,B). A similar correlation between low ALDH expression and a higher capacity to generate mammospheres was also observed in MDA-MB-468 cells (ALDH^Low^ vs. ALDH^High^ cells) (Figure S5). MDA-MB-231 with low ALDH content also showed a greater capacity to generate colonies (21.67 ± 1.59 vs. 7.55 ± 0.60) with a 3-fold higher number of colonies than ALDH^High^ sub-populations after 14 days (Figure 4C,D). 

### 3.4. ALDH^High^ Cells Are Associated with Greater Expression of Senescence Markers

**Figure 5:**
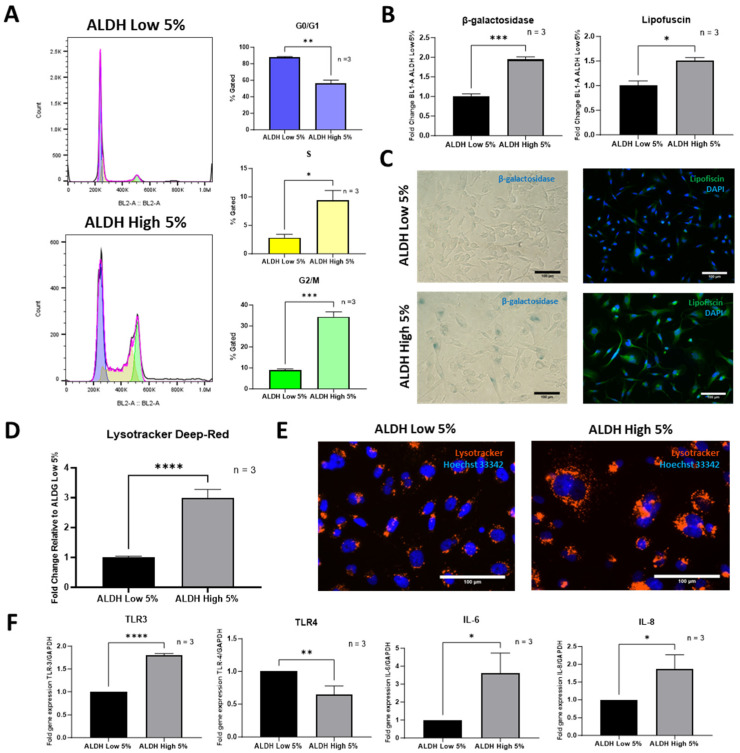
ALDH^High^ MDA-MB-231 cells display a more senescent phenotype than ALDH^Low^ sub-populations. A large segment of ALDH^High^ cells were observed to be in the G2/M phase of the cell cycle, while ALDH^Low^ cells were predominantly observed to be in the G0/G1 phase, as analyzed via flow cytometry (**A**). The senescent/aging markers β-galactosidase and lipofuscin were also observed to be present at a higher degree in ALDH^High^ sub-populations. Senescent/aging markers were analyzed via flow cytometry, and a visual representation is displayed via microscopic imaging (**B**,**C**). The lysosomal mass of ALDH^High^ cells was also shown to be greater than that of ALDH^Low^ sub-populations, as measured by flow cytometry and visualized via the fluorescence imaging of Lysotracker Deep Red (**D**,**E**). Senescence and inflammation genes measured using RT-qPCR were also observed to be increased in ALDH-high populations (*TLR3, IL-6* and *IL-8*) with the exception of *TLR4* (**F**). Experiments were performed at least 3 times independently. Data significance is presented as * *p* ≤ 0.05, ** *p* ≤ 0.01, *** *p* ≤ 0.001 and **** *p* ≤ 0.0001.

Cells that undergo senescence were initially thought to enter an irreversible cell cycle arrest in the G1 phase. However, recent studies have shown that the JNK and p53-p21 signaling pathways mediate the entry of senescent cells into a permanent G2/M arrest. These cells are not only in a senescent state but also secrete pro-inflammatory cytokines/growth factors [35,36,37]. In our study, a larger number of ALDH^High^ MDA-MB-231 cells were in the G2/M (34.40 ± 1.93%) and S phases (9.38 ± 1.43%) of the cell cycle as compared to ALDH^Low^ cells (8.93 ± 0.49% and 2.81 ± 0.48%, respectively). ALDH^Low^ sub-populations were shown to predominantly exist in the G0/G1 phase (88.25 ± 0.44%), whereas there were significantly fewer ALDH^High^ sub-populations in that phase (56.21 ± 3.33%) (Figure 5A). Even larger differences in the number of cells present in the G2/M phase were observed among the MDA-MB-468 ALDH^High^ and ALDH^Low^ cells (Figure S6). In the same line of evidence, ALDH^High^ MDA-MB-231 cells were also shown to exhibit a higher expression of senescence markers. ALDH^High^ sub-populations displayed ~2-fold and ~1.5-fold higher content of β-galactosidase and lipofuscin, respectively, than ALDH^Low^ cells (Figure 5B,C). Furthermore, ALDH^High^ MDA-MB-231 (Figure 5D,E) and MDA-MB-468 cells (Figure S7) were shown to possess a larger lysosomal mass compared to their ALDH^Low^ counterpart. Given the inflammatory property of senescent cells, as demonstrated in various studies, we evaluated numerous inflammatory markers in the MDA-MB-231 sub-populations. Our data showed that cells with high ALDH exhibited a greater gene expression of the toll-like receptor 3 (*TLR3*), which has recently been shown to be linked to senescence [38,39,40] (Figure 5F). However, TLR4 was downregulated in ALDH^High^ MDA-MB-231 cells (Figure 5F). Interestingly, the expression of the inflammatory markers Interleukin 6 (*IL-6*) and Interleukin 8 (*IL-8*) was prominently upregulated in ALDH^High^ sub-populations compared to ALDH^Low^ cells (Figure 5F). To further investigate the inflammatory profile of the ALDH-high population, we performed RNAseq, carried out by Novogene (UK) Company Limited (Cambridge, UK), on the ALDH^High^ vs. low cells (n = 4 biological replicates). Differentially expressed genes (DEGs) are included in Figure S8A. The top four upregulated differentially expressed genes (DEGs), *SAA2, UBE2C, CEMIP* and *ANXA8L1*, have been associated with increased inflammation, cancer progression, metastatic potential and overall poor survival outcomes of various cancers including breast cancer [41,42,43,44]. DO and DisGeNet enrichment analyses outlined associations with various infections/diseases (Figure S8B,C). Interestingly, GO, KEGG and Reactome enrichment analyses highlighted a significant upregulation of inflammatory-linked pathways, including inflammatory response (GO), cytokine–cytokine receptor interaction (KEGG) and Interferon signaling (Reactome) in the ALDH^High^ cells. Taken together, these data suggest that increased levels of ALDH in MDA-MB-231 cells are linked to a pro-inflammatory phenotype.

### 3.5. ALDH^High^ Sub-Populations Have Larger Mitochondrial Mass and Are More Metabolically Active

**Figure 6:**
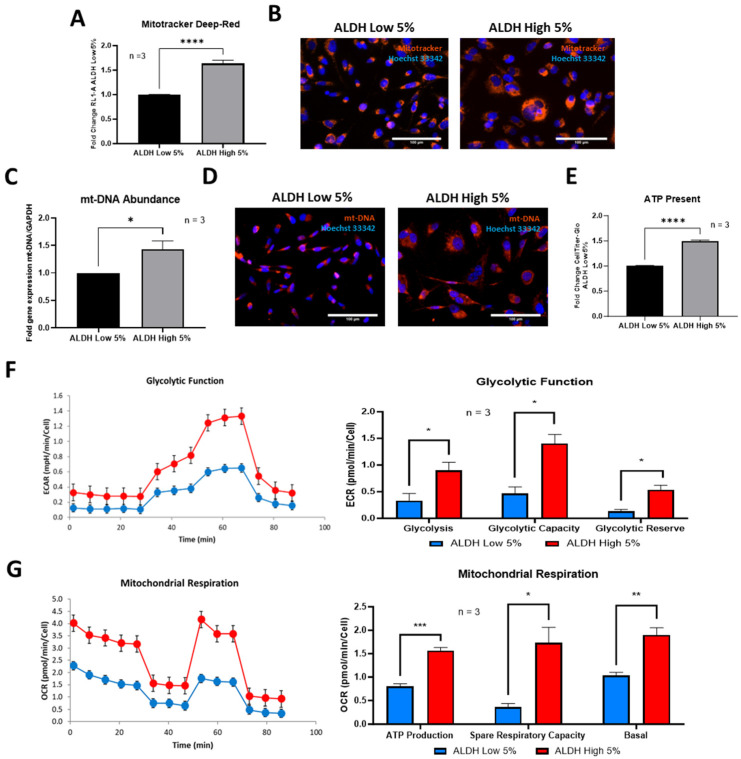
ALDH^High^ MDA-MB-231 cells have greater mitochondrial mass and are more metabolically active than ALDH^Low^ sub-populations. Lysosomal mass was greater in ALDH^High^ sub-populations than ALDH^Low^ sub-populations, as quantified with Lysotracker deep red via flow cytometry and visualized via immunofluorescent imaging (**A**,**B**). ALDH^High^ cells also had a higher abundance of mitochondrial DNA, as measured via qPCR and visualized via immunofluorescence imaging (**C**,**D**). ATP production measured using CellTiter-Glo^®^ 2.0 (**E**) and glycolytic function and mitochondrial respiration parameters measured using Seahorse XFe-96 Metabolic Flux Analysis were also significantly greater in ALDH^High^ sub-populations (**F**,**G**). Experiments were performed at least 3 times independently. Data significance is presented as * *p* ≤ 0.05, ** *p* ≤ 0.01, *** *p* ≤ 0.001 and **** *p* ≤ 0.0001.

As previously mentioned, cancer cells that undertake EMT changes also go through metabolic reprogramming. This metabolic rewriting in CSCs is believed to occur simultaneously with the EMT process and is associated with rapid ATP production and a glycolytic phenotype while retaining functional mitochondria. These changes have also been linked with enhanced cell migration ability and increased stemness [20,21]. In our study, ALDH^High^ sub-populations demonstrated a larger mitochondrial mass (~1.5-fold, Figure 6A,B) and a similar abundance of mitochondrial DNA (mt-DNA) (Figure 6C,D) as compared to ALDH^Low^ cells. Such increased mitochondrial mass was correlated with increased ATP content in ALDH^High^ sub-populations (Figure 6E). ECAR measurements for assessing glycolytic function demonstrated that ALDH^High^ cells exhibited increased glycolysis, glycolytic capacity and glycolytic reserve compared to ALDH^Low^ sub-populations (Figure 6F). OCR differences were also observed in the two ALDH sub-populations, with ALDH^High^ cells showing increased mitochondrial respiration features and higher levels of ATP production of spare respiratory capacity and basal OCRs (Figure 6G).

### 3.6. ALDH^High^ Cells Possess More Migratory Features, While ALDH^Low^ Cells Are More Proliferative

**Figure 7:**
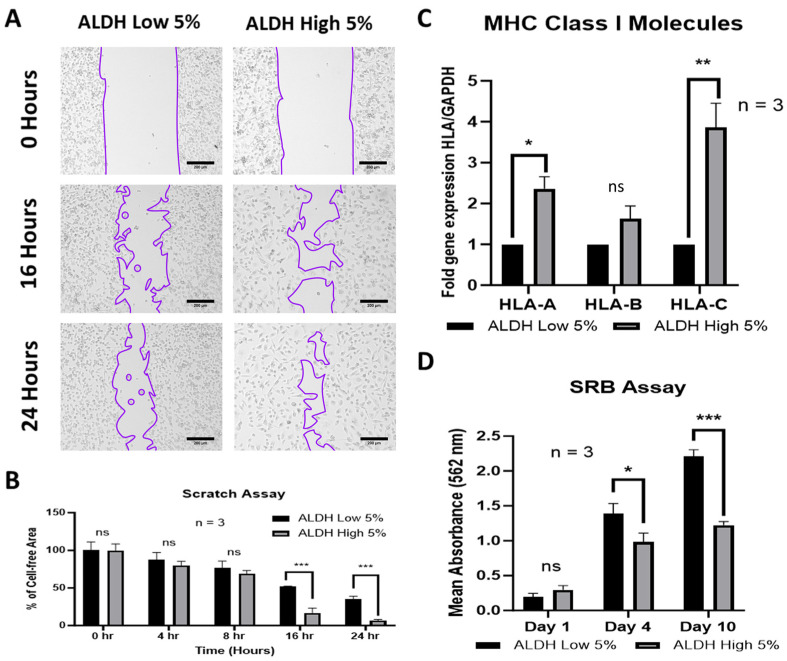
ALDH^High^ MDA-MB-231 cells display more migratory features, while ALDH^Low^ sub-populations are more proliferative. The percentage of cell-free area was significantly reduced in ALDH^High^ cells after 16 and 24 h compared to the ALDH^Low^ sub-populations, as observed via a scratch assay (**A**,**B**). ALDH^High^ cells had a higher gene expression of MHC class I molecules (*HLA-A* and *HLA-C*) compared to ALDH^Low^ cells, with the exception of HLA-B (**C**). ALDH^Low^ sub-populations had greater proliferative ability after 10 days compared to ALDH^High^ sub-populations, as measured by SRB absorbance assay (**D**). Experiments were performed at least 3 times independently. Data significance is presented as * *p* ≤ 0.05, ** *p* ≤ 0.01 and *** *p* ≤ 0.001. Value *p* > 0.05 were considered not significant (ns).

In order to assess the migration potential of cells, a scratch assay was performed on the two ALDH sub-populations. ALDH^High^ cells displayed a higher migration capacity, with the initial gap created by the scratch being significantly repopulated by cells after 16 h as compared to ALDH^Low^ sub-populations. By 24 h, the gap in the layer of ALDH^High^ cells was almost entirely covered by cells (6.72 ± 0.79% cell-free area in ALDH^High^ vs. 34.77 ± 2.41% in ALDH^Low^ cells, Figure 7A,B). In addition to migration, another phenomenon of cancer cells is increased proliferation. To evaluate the proliferative capacity of our cells, we undertook an SRB assay, demonstrating that while ALDH^High^ cells adopted a more migratory phenotype, ALDH^Low^ sub-populations exhibited a greater proliferative potential. After an incubation period of 10 days, ALDH^Low^ cells demonstrated a higher rate of proliferation than ALDH^High^ sub-populations (Figure 7D).

Another feature of the tumorgenicity of cells is their expression of major histocompatibility complex (MHC) class I molecules. MHC class I molecules have immunosuppressive features in MSCs, helping them to avoid natural killer cell-mediated cytotoxicity. However, cancer cells demonstrate a downregulation of their MHC class I molecules, a feature associated with more aggressive tumorgenicity [45,46,47]. In this study, ALDH^High^ cells exhibited a high gene expression of MHC class I molecules (*HLA-A* and *HLA-C*), in concomitance with their capacity to adopt a more mesenchymal phenotype compared to ALDH^Low^ cells, as demonstrated above (Figure 7C).

### 3.7. Azithromycin Shows Anti-Cancer Potential Against MDA-MB-231 Breast Cancer Cells In Vitro

**Figure 8:**
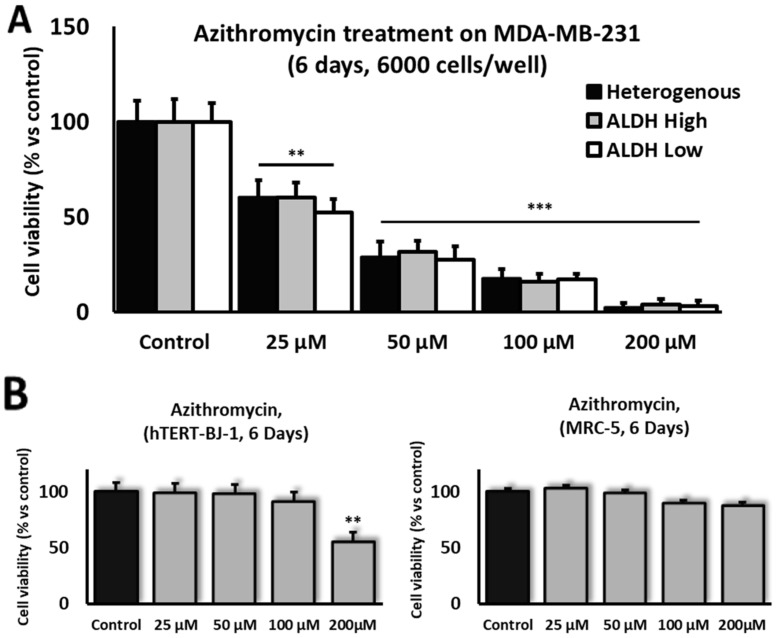
ALDH-sorted MDA-MB-231 cells and unsorted heterogenous populations showed higher sensitivity to Azithromycin than hTERT-BJ-1 or MRC-5 fibroblast cell lines. After 6 days, the cell viability of MDA-MB-231 ALDH-sorted sub-populations and MDA-MB-231 heterogenous populations significantly decreased and reached the lowest concentration (25 µM) compared to untreated controls and continued to decrease further with higher concentrations of Azithromycin (**A**). The cell viability of fibroblast cell lines hTERT-BJ-1 and MRC-5 remained similar to that of the control after 6 days of Azithromycin treatment, with the exception of hTERT-BJ-1 cells, whose cell viability significantly decreased at the highest concentration of 200 µM (**B**). Experiments were performed at least 3 times independently. Data significance is presented as ** *p* ≤ 0.01 and *** *p* ≤ 0.001.

For decades, Azithromycin has become a widely used FDA-approved macrolide antibiotic for treating various types of bacterial infections. However, in recent years, several studies have shown that it possesses anti-cancer effects by targeting mitochondrial activity and inhibiting the autophagy of multiple cancer cell lines [48,49]. In our study, Azithromycin showed efficiency in targeting MDA-MD-231 cells, including both ALDH^High^ and ALDH^Low^ sub-populations, as well as a heterogenous (unsorted) population of MDA-MD-231 cells. The viability of MDA-MB-231 cells was reduced upon their treatment with Azithromycin (for 6 days), even at a low concentration of 25 µM, as compared to hTERT BJ-1 or MRC-5 fibroblast cell lines. Higher concentrations of 50 µM, 100 µM and 200 µM of Azithromycin demonstrated even more effects on MDA-MD-231 breast cancer cell lines vs. fibroblasts (Figure 8A,B).

## 4. Discussion

In the present study, we demonstrated that high levels of ALDH in CSCs have a positive correlation with CSCs’ potential to undertake the EMT process and adopt a mesenchymal phenotype. The sub-population of CSCs with high ALDH content possessed increased levels of stemness markers and senescence characteristics. ALDH^High^ cells also demonstrated higher metabolic activity and migration potential. Conversely, ALDH^Low^ sub-populations displayed a greater proliferative ability and more efficiency in producing mammospheres and colonies. 

The EMT process has been notoriously associated with tumor initiation, metastasis and therapeutic resistance. It is believed to be a key process in the differentiation of epithelial cells into CSCs and considered a major driver of cancer progression [5,6]. To distinguish CSCs in tumors, multiple biomarkers have been identified to differentiate them from other cancer cells. One of these markers is ALDH, a group of enzymes responsible for oxidizing aldehydes and removing toxins from cells to maintain their survival [12,13]. In this study, we demonstrated that CSCs can be selected from a heterogenous breast cancer epithelial cell population using ALDH as a biomarker. Cells segregated for their high ALDH content (ALDH^High^) demonstrated an increased expression of pluripotency markers (Nanog and Sox2), senescence characteristics (G2/M arrest, lysosomal mass, β-galactosidase and lipofuscin expression) and MSCs markers (Vimentin, CD90, CD105), underscoring their high potential for stemness and the mesenchymal transition, i.e., EMT, compared to ALDH^Low^ sub-populations.

Over the last few decades, MSCs have often been praised as favorable candidates for various clinical applications, with studies demonstrating the safety of these applications. This is mainly due to the immunomodulation features of MSCs via the expression of MHC class I molecules which assist them in avoiding immune rejection by the host, protecting them from natural killers [50,51]. However, in recent years, the clinical safety of using MSCs has been disputed. Indeed, while some subsets of MSCs exhibit anti-tumorigenic characteristics [52,53,54], other MSC sub-populations have been shown to contribute to cancer development/progression by migrating into tumor sites and developing cancer-initiating properties. They have been associated with immunosuppressive characteristics and expanded more specifically in metastatic tumors [55]. Moreover, these MSC subsets have been linked with senescence features or late passaging in culture and present an altered immunophenotype from other MSCs, as well as higher OCRs [56,57]. In our study, we showed that ALDH^High^ cells not only seemed to possess more mesenchymal phenotype characteristics but also displayed increased senescence. In recent years, it has been discovered that cells resistant to chemo/radiotherapy can develop therapy-induced senescence with increased stemness and ALDH expression. Furthermore, therapy-induced senescent cells can acquire a senescence-associated secretory phenotype (SASP), which can contribute to a pro-tumorigenic environment. These cells have been characterized by increased lysosomal activity, including that of senescence-associated lysosomal enzyme β-galactosidase and lysosomal lipoprotein aggregates like lipofuscin, and do not proliferate or have low cell division potential. However, SASP cells have also been demonstrated to remain metabolically active and secrete various biologically active substances such as growth factors like vascular endothelial growth factor (VEGF), connective tissue growth factor (CTGF) and insulin-like growth factor-binding proteins (IGFBPs). The secretion of these agents can alter microenvironments to promote the active proliferation of neighboring cells and is believed to play a critical role in tumor progression, metastasis, angiogenesis and therapy resistance [58,59]. Furthermore, our data demonstrating the inflammatory signature of our ALDH^High^ CSCs are in accordance with those of previously described studies outlining the capacity of CSCs to secrete the pro-inflammatory cytokines TLRs (*TLR3*) and ILs (*IL-6* and *IL-8*), and genes associated with cell aging and inflammation were also upregulated in ALDH^High^ sub-populations. In our study, *TLR4* was surprisingly downregulated in ALDH^High^ cells. Indeed, the upregulation of *TLR4* has been associated with increased breast cancer metastasis and a high proliferative activity and cell senescence of tumors. *TLR4* is also known to be upregulated in cells secreting pro-inflammatory factors such as *IL-6* or *IL-8* [60,61]. Our result showing that *TLR4* was downregulated in ALDH^High^ cells contradicts the previously shown correlation of TLR4 with cancer development and metastasis, suggesting that *TLR4* may have a regulatory role in cancer development through the modulation of cell proliferation and tumor growth, as opposed to cell migration or metastasis. Although the study of the specific role of *TLR4* is outside the scope of the current study, determining the significance of *TLR4* levels and the conflicting roles in tumor progression opens up an interesting avenue for further investigation.

ALDH^High^ sub-populations also displayed larger mitochondrial mass and activity and showed a migratory phenotype, similarly to the tumorigenic MSC subsets detailed above. Altogether, these findings suggest that the senescent/pro-tumorigenic MSC subsets identified in various studies over the years can potentially be cancer epithelial cells that have undergone the EMT process and adopted various MSC or MSC-like features like those reported in this study.

To isolate CSCs, various studies have used CD44 and ALDH as key biomarkers. Indeed, a high expression of ALDH and CD44 has often been associated with CSCs. The ALDEFLUOR™ kit used to isolate CSCs in this study has previously been shown to select cells expressing high levels of ALDH1A1, ALDH3A1, ALDH1A3 and ALDH2 isoforms, which are closely linked to breast cancer stem cells [62,63,64]. In line with our results shown here, cells isolated using these biomarkers have been correlated with increased vimentin expression and stemness potential [7,8,18]. Vimentin and ALDH expression has also been linked to greater efficiency in producing mammospheres and colonies [9,19]. With respect to this latter characteristic, our data, on the contrary, showed that ALDH^High^ cells were less efficient in generating mammospheres and colonies, despite overexpressing stemness markers. ALDH^Low^ sub-populations exhibited higher proliferative potential, which might be a reason for their superior capacity at generating mammospheres and colonies in our study. Another interesting characteristic observed in cancers and tumor cells is the altered expression of MHC molecules, presenters of antigens to effector immune cells. MHC class I molecules have been shown to be decreased in cancer cells as a mechanism of evading the immune system to promote tumor progression, as well as migration/invasion [65,66]. However, in MSCs, in spite of evidence pointing to their low MHC content responsible for their low immunogenicity, some studies have shown opposite results in that the high expression of MHC class I in MSCs is usually associated with their survival against the immune system, more specifically against natural killer cell cytotoxicity [46]. Interestingly, our results herein are in line with this latter notion and demonstrated that CSCs with high ALDH content, shown to have a mesenchymal phenotype, displayed an upregulated expression of MHC class I genes compared to ALDH^Low^ cells. As a further validation of the invasiveness of our CSCs, very likely undertaking an EMT process, we outlined their increased capacity to migrate. This contradicts recent studies related to the downregulation of MHC class I genes in cancer cells and the immunoregulatory features of MSCs which suggest that the expression of MHC class I genes is key to escaping the immunosurveillance of natural killer cells [45,46,47,50]. This area needs further exploration to gain a more conclusive understanding of the role of MHC class I molecules in the immunomodulation of cancer cells and more specifically of CSCs and MSCs.

In this report, we also investigated the potential therapeutic effects of Azithromycin on breast CSCs. Over the last few years, several studies have demonstrated that Azithromycin possesses anti-cancer properties by targeting mitochondria and autophagy mechanisms in tumor cells [48,49,67]. Azithromycin was shown to inhibit cell survival and the proliferation of several cancer cell lines derived from breast (MCF-7), lung (A549) or colon cancer (SW480 and SW620) [49,67,68]. Our data further validate the therapeutic potential of this antibiotic in tumors by demonstrating its anti-cancer effect on MDA-MB-231 cells, decreasing their viability. Interestingly, Azithromycin is also known for its effect on the mesenchymal transition of airway epithelial cells, a crucial process in the pathology of numerous airway diseases, such as asthma, chronic obstructive pulmonary disease and lung cancer [69,70]; thus, its influence on the EMT process of CSCs is highly likely and should be more deeply investigated in this context.

## 5. Limitations

While this study provides valuable insight into various aspects of ALDH in CSCs, certain limitations of this research should be acknowledged. The reliance on in vitro models does not fully replicate the in vivo environment, and various details of these findings may not be fully translational in such a complex setting. CSCs demonstrate a variety of markers across cancer cell types, as well as different forms of cancers. While we demonstrated that the two different breast cancer cell lines used exhibit some of the same features when sorted for their ALDH content, similar findings may vary when compared to cell lines derived from other tumorigenic tissues. While it is promising that Azithromycin was demonstrated to be able to target both ALDH sub-populations, the mechanisms behind this must be investigated further. The results of this study should be reviewed, taking these limitations into account, and additional studies are required to better understand some of the mechanisms related to proliferation and enhanced mammosphere generation from ALDH^Low^ sub-populations and the relationship that inflammation/senescence has to ALDH^High^ sub-populations. Future work should focus on better understanding these mechanisms and identifying which ALDH isoforms are more highly expressed in these sub-populations. Lastly, in vivo experiments should be conducted to validate the mesenchymal/migratory phenotype that ALDH^High^-sorted cells displayed and obtain a greater understanding of their metastatic potential.

## 6. Conclusions

In summary, breast cancer stem cells expressing high ALDH demonstrated MSC-like characteristics associated with EMT with enhanced stemness potential and migratory ability. Furthermore, these cells exhibited increased levels of senescent biomarkers than ALDH^Low^ sub-populations, including β-galactosidase and lipofuscin; a larger lysosomal mass; and upregulated inflammatory genes. Moreover, ALDH^High^ sub-populations had a greater expression of MHC class I molecules which are known to be important regulators of immunogenicity. Nevertheless, ALDH^Low^ sub-populations still demonstrated a higher capacity for proliferation and the generation of mammospheres and colonies than ALDH^High^ sub-populations. This study supports the fact that these ADLH sub-populations possess significant differences, yet both can be targeted by Azithromycin in vitro at doses that are not cytotoxic to multiple non-cancerous fibroblasts, opening up interesting avenues for further investigation.

## Data Availability

The original contributions presented in this study are included in this article/Supplementary Materials; further inquiries can be directed to the corresponding authors.

## Supplementary Materials

The following supporting information can be downloaded at https://www.mdpi.com/article/10.3390/cells13242059/s1, Figure S1: Primary antibodies used for flow cytometry; Figure S2: RT-qPCR list of primers and melting curves; Figure S3: FITC ALDH sorting MDA-MB-468; Figure S4: RFP vimentin sorting MDA-MB-231; Figure S5: MDA-MB-468 mammospheres; Figure S6: MDA-MB-468 cell cycle; Figure S7: MDA-MB-468 Lysotracker red; Figure S8: RNA seq data.
