# Supplementary material for: ALDHHigh Breast Cancer Stem Cells Exhibit a Mesenchymal–Senescent Hybrid Phenotype, with Elevated Metabolic and Migratory Activities
Source: Cells. 2024 Dec 13;13(24):2059. doi: 10.3390/cells13242059 (PMC11674378; doi:10.3390/cells13242059)

Supplemental

Figure S1 primary antibodies used for Flow Cytometry

Phenotype	Marker	Fluorophore	Supplier and Code	
**Pluripotent**	OCT 4	PE	Sony	3868520
	SOX 2	Unconjugated	Abcam	ab93689
	NANOG	Alexa 647	Sony	3971050
**EMT/MSC**	CD90	FITC	BD bioscience	11-0909-42
	CD73	APC-780	BD bioscience	47-0739-42
	CD105	PE	BD bioscience	17-1057-42
	CD44	APC	BD bioscience	17-0441-82

List of primary antibodies used for Flow Cytometry analysis

Figure S2 RT-qPCR Primers and Melting curve

Target Gene	Primer Sequence
**hTLR3**	Forward: 5′-ACACACTTCCAGCATCTGTC-3′
	Reverse: 5′-TCACACGTGCAATCAAAGGG-3′
**hTLR4**	Forward: 5′- TGGGAGCCTTTTCTGGACTA-3′
	Reverse: 5′-TGGAAAGGTCCAAGTGCTCT-3′
**hIL-6**	Forward: 5′-CAGAGCTGTGCAGATGAGTA-3′
	Reverse: 5′-CTCCTTAAAGCTGCGCAGAA-3′
**hIL-8**	Forward: 5′-GTGCAGTTTTGCCAAGGAGT-3′
	Reverse: 5′- AACCCTCTGCACCCAGTTTT-3′
**HLA-A**	Forward: 5′-TCCTTGGAGCTGTGATCACT-3′
	Reverse: 5′- AAGGGCAGGAACAACTCTTG -3′
**HLA-B**	Forward: 5′-ATTACATCGCCCTGAACGAG-3′
	Reverse: 5′-ATCTCCGCAGGGTAGAAACC-3′
**HLA-C**	Forward: 5′-TCCTGGTTGTCCTAGCTGTC-3′
	Reverse: 5′-CAGGCTTTACAAGTGATGAG-3′
**GAPDH**	Forward: 5′-CCCACTAACATCAAATGGGG-3′
	Reverse: 5′-CCTTCCACAATGCCAAAGTT-3′

List of primers used for RT-qPCR analysis

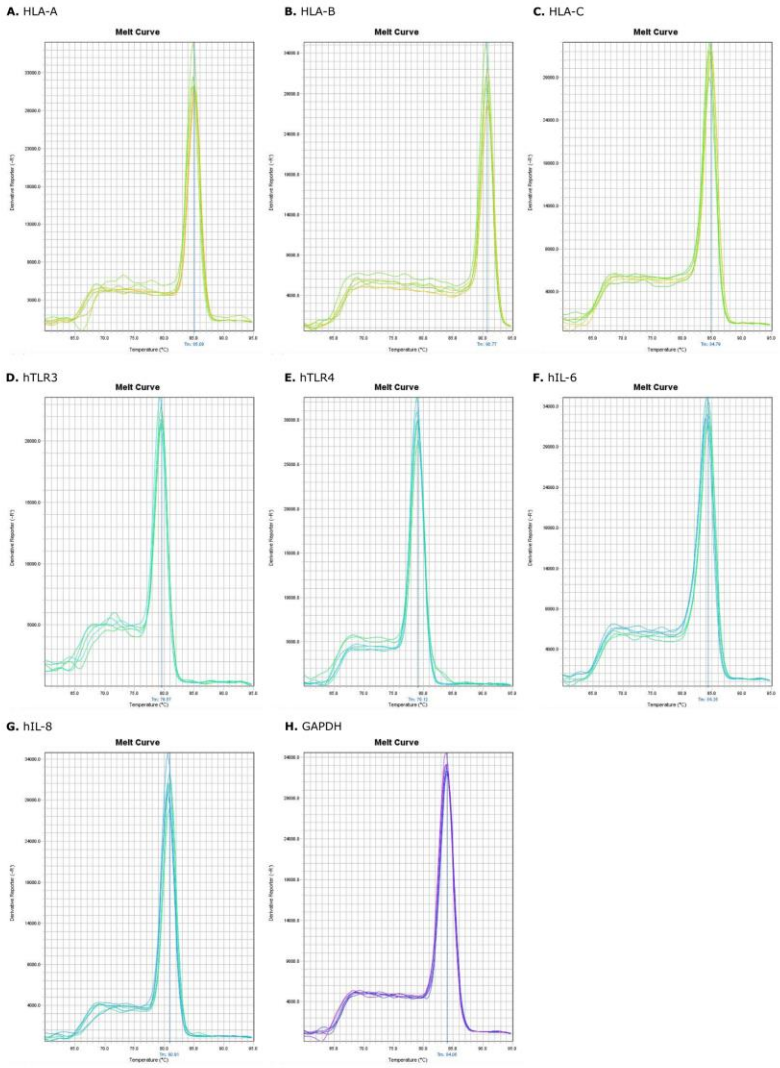

Figure S3 FITC ALDH sorting MDA-MB-468

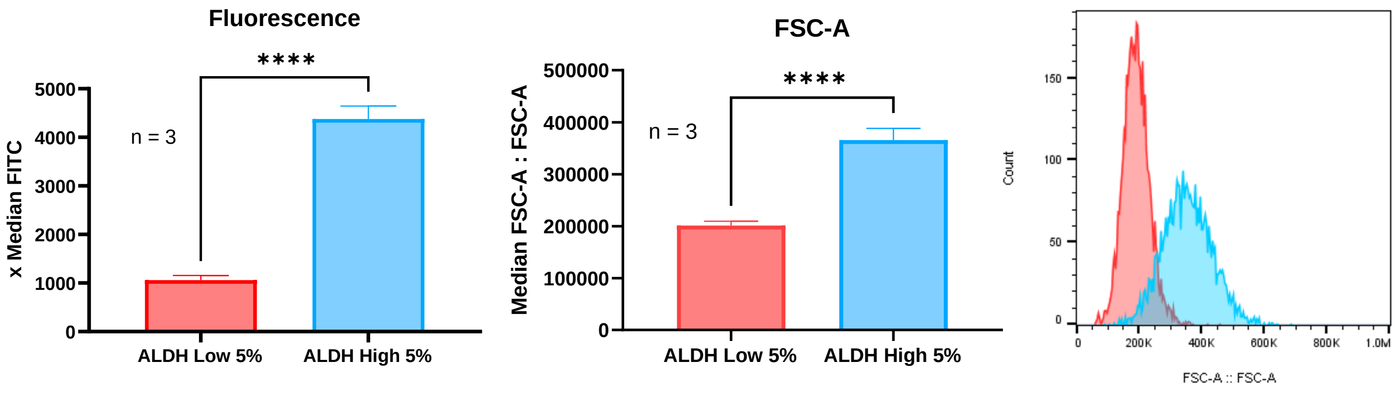

Figure S4 RFP Vimentin sorting MDA-MB-231)

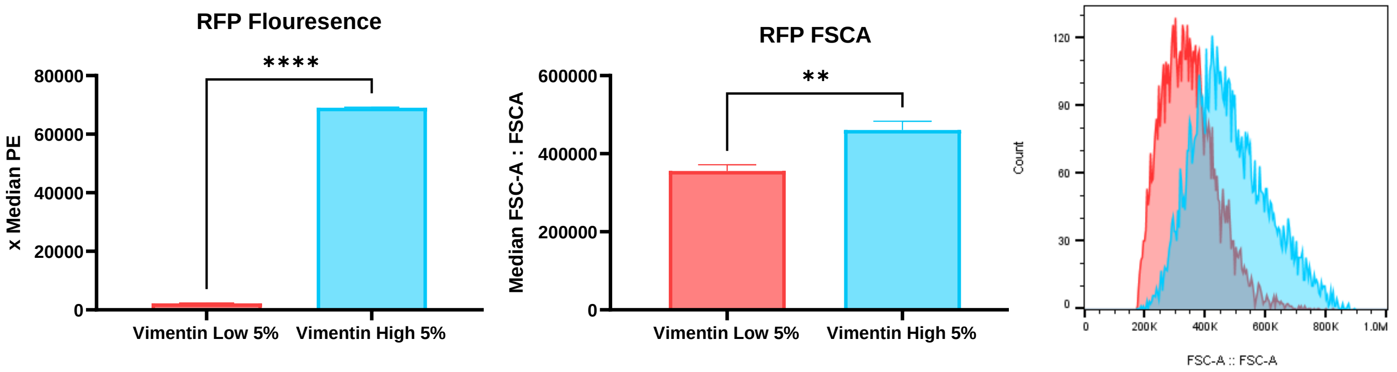

Figure S5 Mammospheres MDA-MB-468

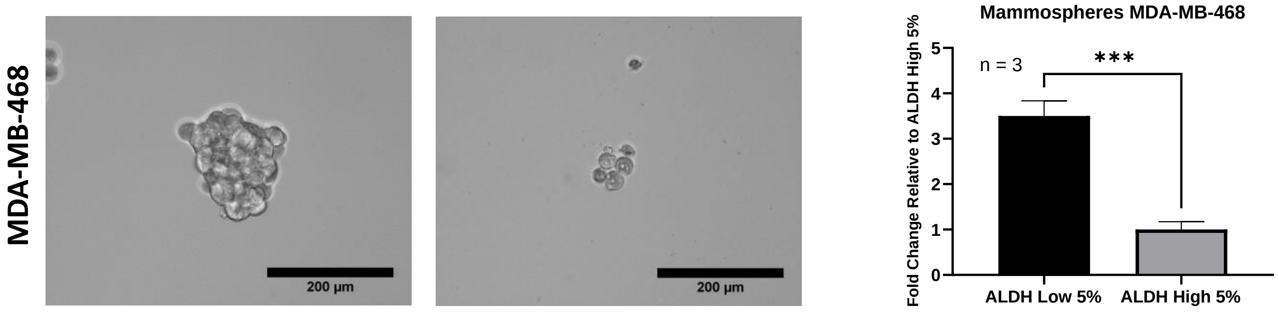

Figure S6 Cell Cycle MDA-MB-468

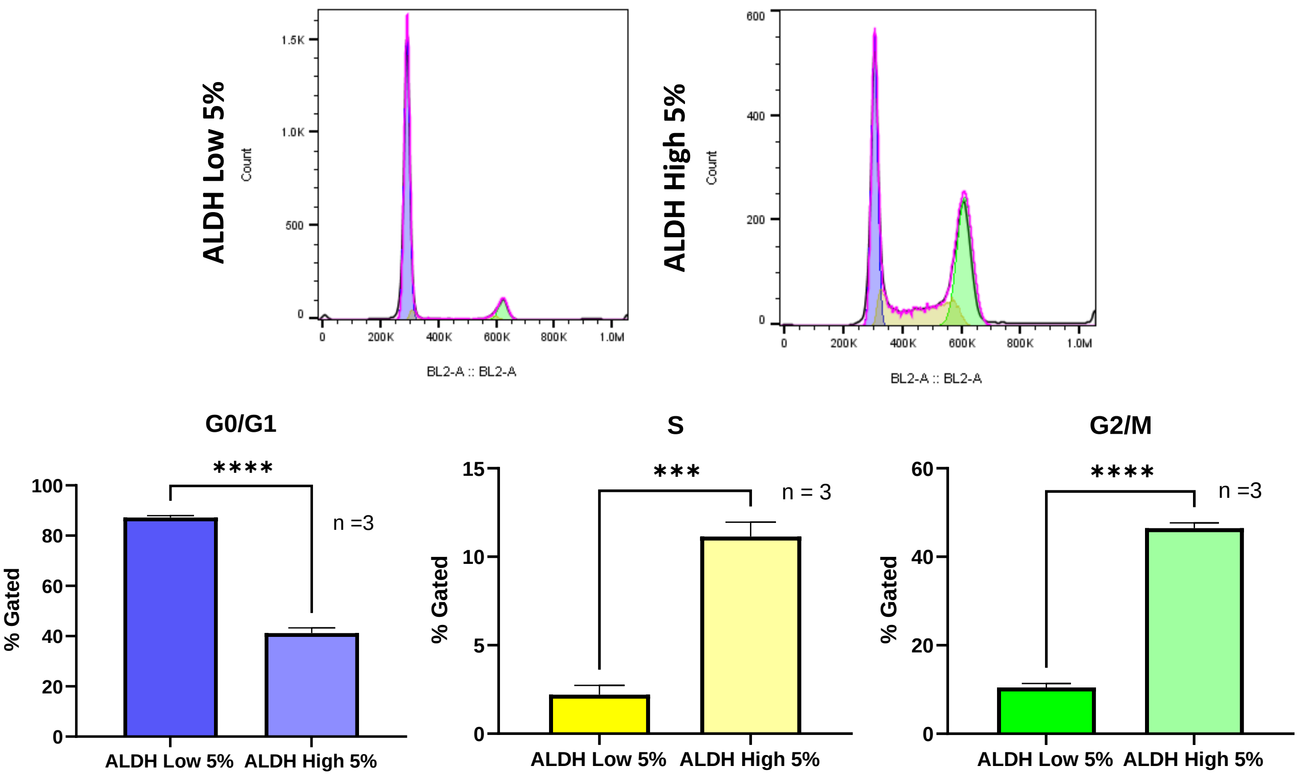

Supplemental 7 (Lysotracker red MDA-MB-468)

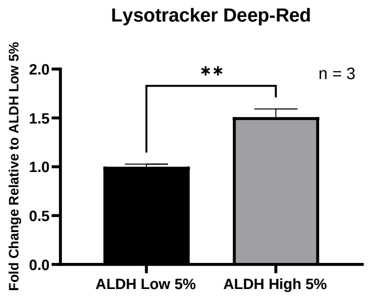

Figure S8 RNA seq Data

A

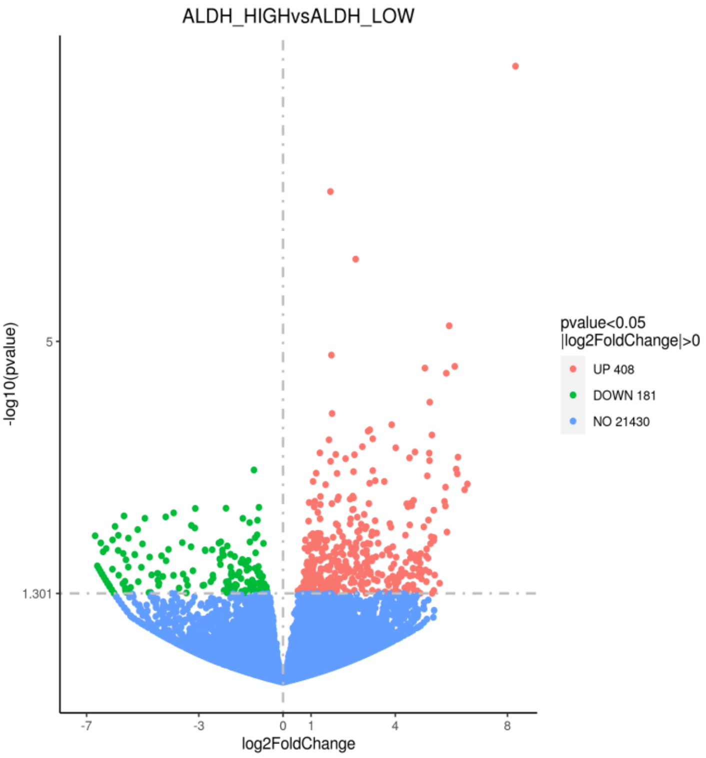

Upregulated DEGs

log2FoldCh	pvalue	padj	gene_name
8.284603	9.15E-10	2.00E-05	SAA2
1.68926	6.33E-08	0.000692	UBE2C
2.589034	6.21E-07	0.004519	CEMIP
5.921715	5.90E-06	0.032228	ANXA8L1
1.725117	1.59E-05	0.069448	CDK1
6.120344	2.33E-05	0.076989	PDZK1IP1
5.057611	2.47E-05	0.076989	SORCS2
5.818748	2.94E-05	0.080159	PLEKHS1
5.229763	7.80E-05	0.189303	HLA-F
1.74765	0.000115	0.250333	HLA-B

B

DisGenNet

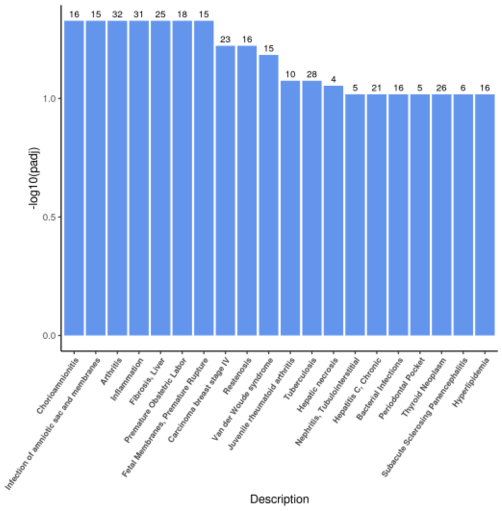

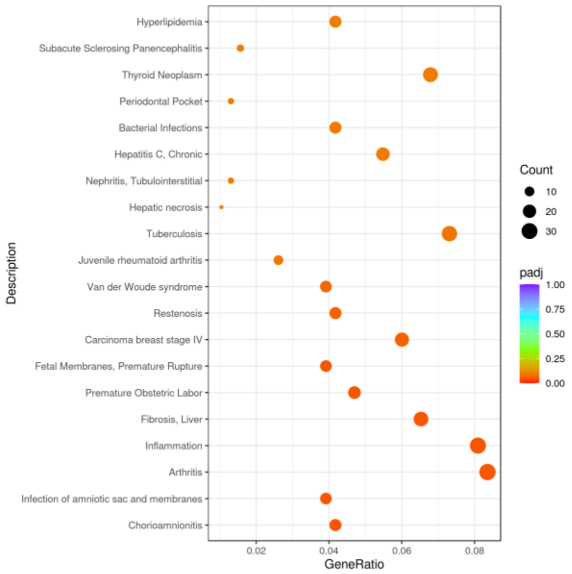

C

DO

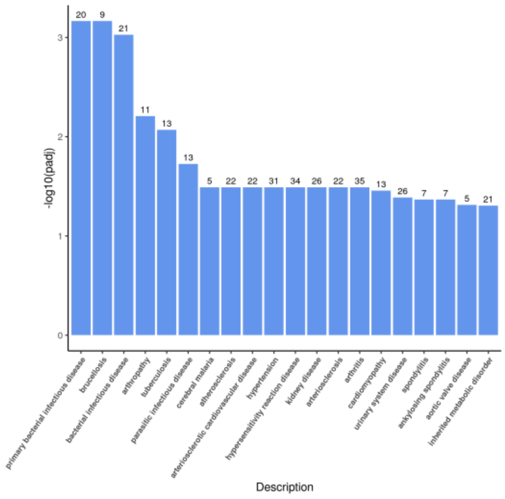

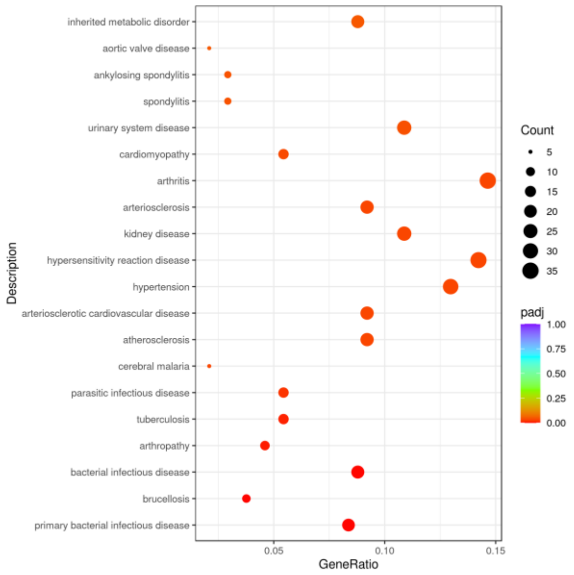

D

GO

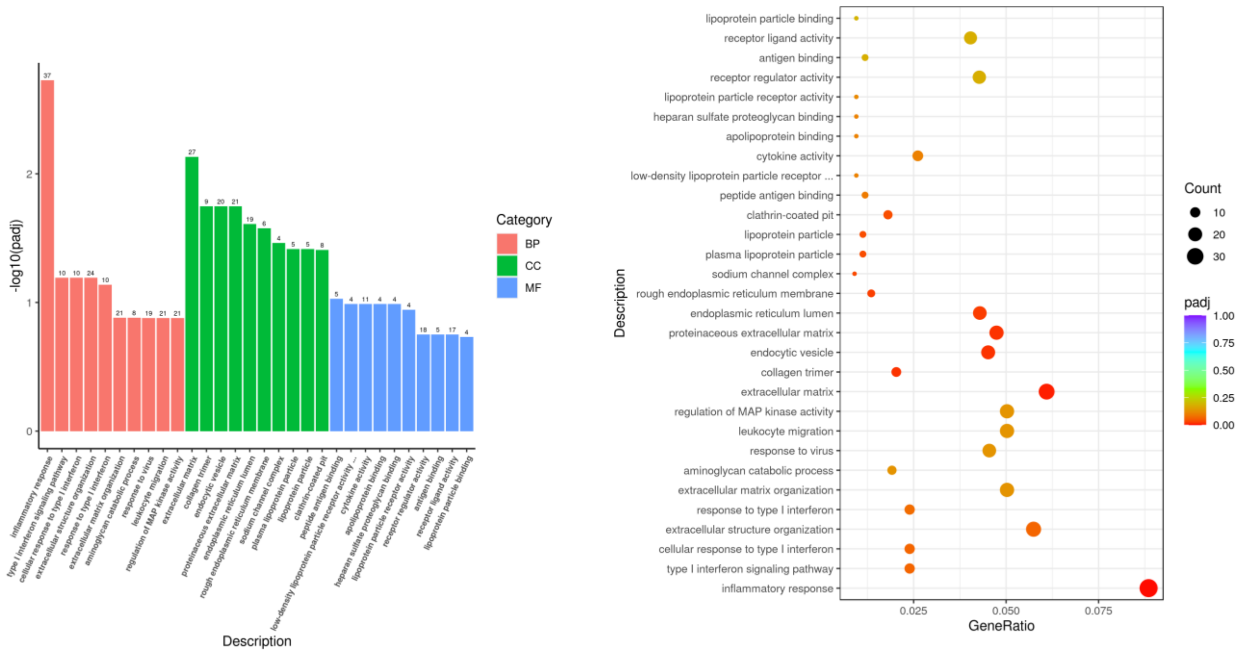

E

KEGG

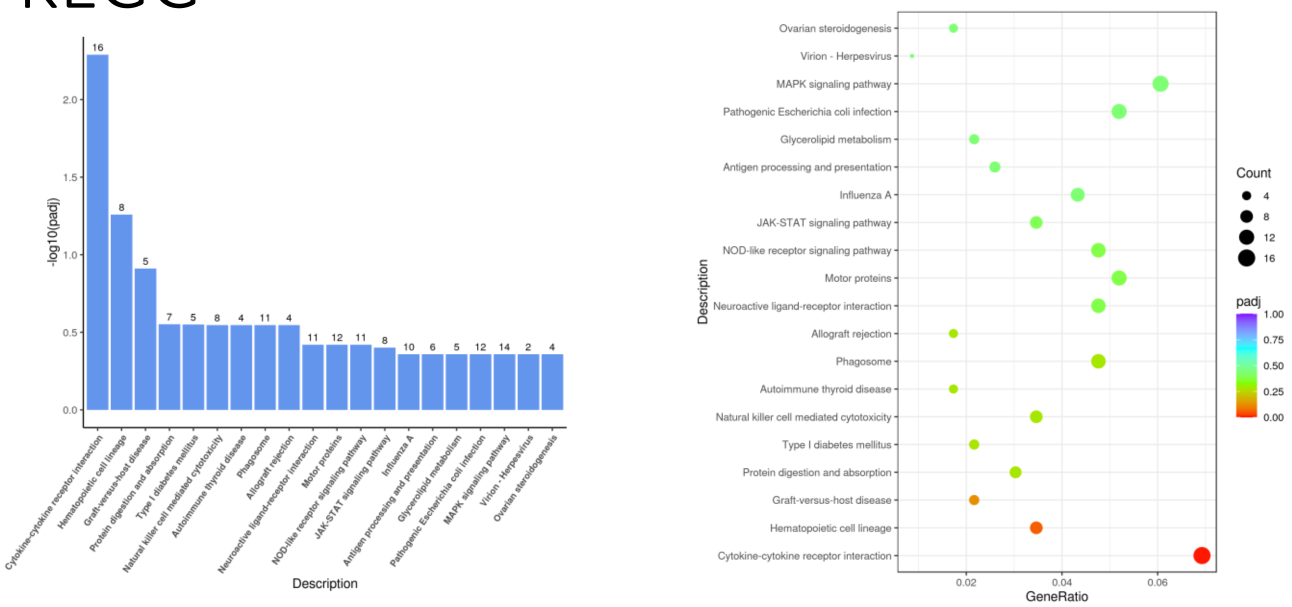

F

Reactome

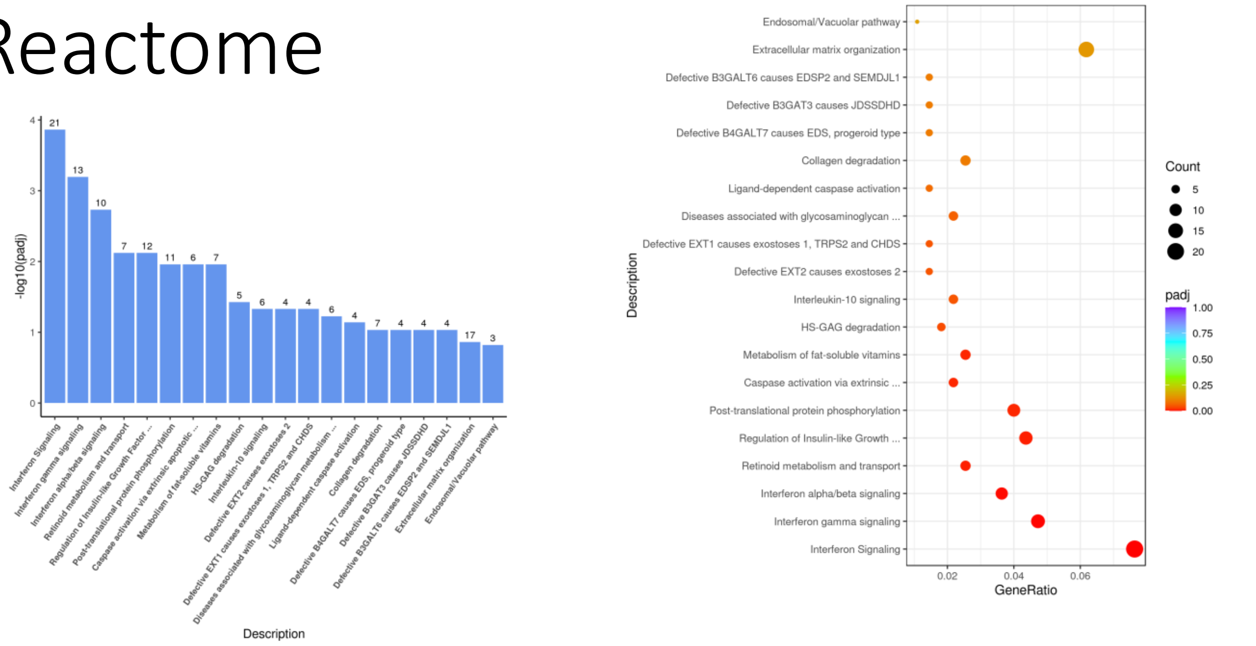

Supplement: Supplementary file 1 [file cells-13-02059-s001.zip › cells-3305650-supplementary.pdf]
